# Elraglusib Induces Cytotoxicity via Direct Microtubule Destabilization Independently of GSK3 Inhibition

**DOI:** 10.1158/2767-9764.CRC-24-0408

**Published:** 2024-11-25

**Authors:** Josh T. Coats, Shuyu Li, Tomoyuki U. Tanaka, Sudhir Tauro, Calum Sutherland, Adrian T. Saurin

**Affiliations:** 1 Division of Cellular Medicine, School of Medicine, University of Dundee, Dundee, United Kingdom.; 2 Division of Molecular, Cell and Developmental Biology, School of Life Sciences, University of Dundee, Dundee, United Kingdom.; 3 Division of Molecular and Clinical Medicine, School of Medicine, University of Dundee, Dundee, United Kingdom.

## Abstract

Elraglusib (9-ING-41) is an ATP-competitive inhibitor of glycogen synthase kinase-3 (GSK3) with preclinical studies demonstrating broad activity against many tumor types. Promising early-phase clinical trial data led to FDA orphan drug status, and a randomized phase II study in combination with cytotoxic chemotherapy in pancreatic cancer has recently completed its recruitment. Similarly, single-agent responses in adult T-cell leukemia/lymphoma and melanoma and combination treatment data in several other tumor types have been encouraging. The elraglusib mechanism of action is unknown, but it is unlikely to act through GSK3 inhibition because cytotoxicity is observed below the IC_50_ for GSK3, and other small molecule GSK3 inhibitors do not produce cytotoxic effects, at least in lymphoma cells. We show here that elraglusib perturbs chromosomal alignment to cause a mitotic arrest in multiple tumor lines. This arrest is caused by direct microtubule (MT) depolymerization, which prevents the attachment of kinetochores to MTs. At clinically relevant doses, these mitotically arrested cells eventually undergo mitotic slippage, leading to gross chromosome missegregation, DNA damage, and apoptosis. These effects explain the cytotoxicity of elraglusib because temporarily pausing cell-cycle progression with the CDK4/6 inhibitor palbociclib abolishes any drug-induced genotoxicity and apoptosis. In summary, elraglusib acts as a direct MT destabilizer both *in vitro* and across multiple cancer types, resulting in mitotic arrest, DNA damage, and apoptosis. These effects likely account for its broad pan-cancer activity, which does not rely upon GSK3 inhibition as they are not replicated by other GSK3 inhibitors.

**Significance:**

Elraglusib was designed as a GSK3 inhibitor and is currently in clinical trials for several cancers. We show conclusively that the target of elraglusib that leads to cytotoxicity is MTs and not GSK3. This has significant implications for ongoing clinical trials of the compound and will help in understanding off-target side effects, inform future clinical trial design, and facilitate the development of biomarkers to predict response.

## Introduction

Glycogen synthase kinase-3 (GSK3) is a ubiquitously expressed serine/threonine kinase critical for many cellular processes, including proliferation and apoptosis (1–3). The two GSK3 paralogs, α and β, are expressed from independent genes and are 98% homologous within the catalytic domain (4). Higher GSK3 expression has been associated with poorer outcomes in some cancers, and it has, therefore, been proposed as a potential therapeutic target (5, 6).

Elraglusib (9-ING-41), developed as an ATP-competitive small molecule GSK3β inhibitor, has demonstrated preclinical efficacy against a wide variety of tumor types, including lymphoma, pancreas, ovarian, bladder, renal, breast, and glioblastoma (6–13). Early evidence of clinical efficacy led to FDA orphan drug status for pancreatic cancer, and a randomized phase II trial in combination with gemcitabine and nab-paclitaxel has recently completed recruitment (NCT03678883). Phase I and case-study data with elraglusib monotherapy also suggest potential efficacy in adult T-cell leukemia/lymphoma and melanoma (14, 15).

We have previously shown that the anti-lymphoma effects of elraglusib occur below the GSK3α/β IC_50_, cannot be replicated with other structurally distinct small molecule GSK3 inhibitors, and are unaffected by *GSK3A/B* knockdown, thereby questioning the role of GSK3 in its mechanism of action (16, 17). We set out to establish the mechanism of action of elraglusib because this will enhance clinical trial design and may improve the development of its anticancer efficacy.

## Materials and Methods

### Compounds and antibodies

Doxorubicin and elraglusib (9-ING-41) were purchased from Cambridge Bioscience, palbociclib from MedChemExpress, LY2090314, nocodazole, and paclitaxel (Taxol) from Stratech, MG132, puromycin, and neomycin from Sigma-Aldrich, 4,6-diamidino2-phenylindole (DAPI) from Thermo Fisher Scientific, and RO3306 from Tocris. CT99021 was synthesized locally as previously described (18). Compounds were dissolved in DMSO before use. Flow cytometry antibody: rabbit anti–phospho-histone 3 (Ser10; Cell Signaling Technology, 3465, RRID: AB_10695860) used at 1:100. Immunoblot antibodies: rabbit total GSK3 (Cell Signaling Technology, 5676, RRID: AB_10547140) rabbit anti–β-tubulin (Cell Signaling Technology, 2128, RRID: AB_823664), rabbit anti-PARP (Cell Signaling Technology, 9542, RRID: AB_2160739), rabbit anti–phospho-histone H2A.X (γH2AX; Cell Signaling Technology, 9718, RRID: AB_2118009), and rabbit anti–β-catenin (Cell Signaling Technology, 9562, RRID: AB_331149) were all used at 1:1,000. Mouse anti-actin (Sigma-Aldrich A3853, RRID: AB_262137) was used at 1:2,000. Immunofluorescence (IF) antibodies: mouse anti–α-tubulin (Sigma-Aldrich T5168, RRID: AB_477579) was used at 1:2,500. Guinea pig anti-CENPC (MBL PD030, RRID: AB_10693556) was used at 1:5,000. Mouse anti-MAD1 (Merck Millipore MABE867, RRID: AB_2910099) was used at 1:1,000. Secondary antibodies for immunoblot: goat anti-rabbit IRDye 800CW (LI-COR 926-32211, RRID: AB_621843) and goat anti-mouse Alexa Fluor 680 (Invitrogen A21057, RRID: AB_2535723), both used at 1:10,000. Secondary antibodies for IF: chicken anti-mouse Alexa Fluor 488 (Invitrogen A21200, RRID; AB_2535786), donkey anti-rabbit Alexa Fluor 568 (Invitrogen A10042, RRID: AB_2534017), and goat anti-guinea pig Alexa Fluor 647 (Invitrogen A21450, RRID: AB_2535867), all used at 1:1,000.

### Cell culture

Karpas-299 (K299; RRID: CVCL_1324) cells were purchased from Public Health England, and MCF7 (RRID: CVCL_0031) and HH (RRID: CVCL_1280) cells were from ATCC. U2OS H2B-GFP/mCherry-α-tubulin were a gift from Rob Wolthuis and were generated as described previously (19). Suspension cells were cultured in RPMI 1640 (Gibco) medium containing glycine supplemented with 10% FBS (Sigma) and 100 U/mL penicillin and 100 μg/mL streptomycin (Gibco). Adherent cells were cultured in DMEM (Gibco) supplemented with 10% FBS (Sigma) with 100 U/mL penicillin and 100 μg/mL streptomycin (Gibco). All cells were maintained at 37°C in an atmosphere of 5% CO_2_. Cells were screened for *Mycoplasma* infection every 2 months by PCR and confirmed to be *Mycoplasma*-free.

### Lentiviral short hairpin RNA GSK3 knockdown

Kill curves were performed with puromycin and neomycin in wild-type (WT) K299 cells using the MTS assay to establish the concentrations to be used in the selection medium. Puromycin (2 μg/mL) and 400 μg/mL neomycin were chosen as these effectively reduced cell viability. Cells were pelleted and resuspended in RPMI 1640 with 10% FBS without penicillin/streptomycin. Cells were counted using a hemocytometer and diluted to a concentration of 10^6^/mL. A 2 mL volume of this suspension was added to each well of a six-well plate. Lentiviruses containing a puromycin resistance cassette for single-paralog GSK3 knockdown were purchased from Sigma. *GSK3A* knockdown: pLKO.1 vector, Pol II cassette 1: PGK-PuroR, Pol III promoter: constitutive hU6, Pol III insert: TRCN0000195223, target sequence: CGG​GTG​TAA​ATA​GAT​TGT​TAT. A further lentivirus containing a neomycin resistance cassette for *GSK3B* knockdown in *GSK3A* knockdown cells (to produce dual paralog knockdown) was purchased from Sigma. *GSK3B* knockdown (pLKO.1 vector, Pol II cassette 1: PGK-NeoR, Pol II promoter: CMV Pol III promoter: constitutive hU6, Pol III insert: TRCN0000010551, target sequence: CAC​TGG​TCA​CGT​TTG​GAA​AGA).

A 5 µL volume of virus suspension was added to each well containing 2 × 10^6^ cells in RPMI/FBS without penicillin/streptomycin. The cells were then incubated for 24 hours. The cell suspensions were transferred into 15 mL Falcon tubes and centrifuged at 300 *g* for 5 minutes. The supernatant was poured off, and the cells were resuspended in complete medium (CM). The cells were then incubated for 72 hours, prior to aspiration into 15 mL Falcon tubes and centrifuged again at 300 *g* for 5 minutes. The supernatant was poured off, and the cells were resuspended in selection medium (CM with 2 μg/mL puromycin) into six-well plates for 24 hours. *GSK3A* KD cells were then infected with the *GSK3B* neomycin short hairpin RNA (shRNA) lentivirus prior to being exposed to neomycin-containing medium (CM with 400 μg/mL neomycin) for 7 days (with medium changes every 48 hours) due to the slower killing kinetics of this agent.

Once at sufficient density, the cells were transferred into T25 flasks in CM. Alternate passages with selection medium were continued indefinitely. Knockdown efficiency was quantified by total GSK3 immunoblot using WT cells as controls.

### Primary human T-cell isolation and proliferation

Primary human T cells were isolated from peripheral blood mononuclear cells (PBMC) taken from healthy volunteers (two male and one female) following written informed consent. Exclusion criteria were as follows: inability to provide informed consent, requiring regular medication, active pregnancy, or blood-borne viral infection. The study was performed in accordance with the Declaration of Helsinki, and ethical approval was provided by the University of Dundee School of Medicine and Life Sciences Research Ethics Committee (application number—UOD-SMED-SLS-RPG-2024-24-02). PBMCs were isolated from venous blood drawn into sodium heparin vacutainer tubes (BD Biosciences) using SepMate tubes (STEMCELL Technologies) and T cells purified using a magnetic activated cell sorting negative selection Pan T cell Isolation Kit (Miltenyi Biotec) as per the manufacturers’ instructions. Briefly, Ficoll-Paque PLUS density gradient medium (Cytiva) was pipetted into SepMate tubes before the addition of blood [diluted in PBS (1:1 v/v)]. Following centrifugation and PBS washes, the pelleted PBMCs were counted, and the appropriate volume of antibody and microbead cocktails was added prior to magnetic activated cell sorting. T cells were then cultured as per other suspension cells (see above).

Rapid-Act T Cell Activation Kit (human, anti-CD3/CD28; Cell Signaling Technology, 88179) was used to induce T-cell proliferation as per the manufacturer’s instructions. Briefly, T cells were counted, and the appropriate volume of Rapid-Act CD3/CD28 complex was added. Activated T cells were either incubated until actively proliferating for tubulin experiments or immediately plated for cell viability assay experiments. RealTime-Glo MT (Promega) cell viability assay was used to compare unstimulated and stimulated T cells as per the manufacturer’s instructions. Briefly, 30,000 cells were added per well of a white-walled 96-well plate (Thermo Fisher Scientific) in 100 μL CM. RealTime-Glo MT reagents were added (1:1,000), and the plate was returned to the incubator for 10 minutes before a baseline luminescence read and then read every 24 hours for 120 hours. Medium-only wells were used to subtract background luminescence. Three individual experiments were performed using T cells from three separate volunteers, and each experimental condition was done in technical triplicate. Data were normalized to the control peak luminescence for unstimulated and stimulated groups.

### Flow cytometry

Four million cells/well were treated for 24 hours in six-well plates and then fixed in ice-cold 70% ethanol for 20 minutes at 4°C. Cells were permeabilized by resuspension in 1 mL 0.25% Triton-X100 in PBS on ice for 15 minutes. Following PBS washes, cells were resuspended in anti–phospho-H3 antibody for 1 hour in the dark and then washed in 1% BSA in PBS and then PBS. Cells were resuspended in 50 μL ribonuclease A (100 μg/mL; Thermo Fisher Scientific) in PBS and 300 μL propidium iodide (50 μg/mL; Thermo Fisher Scientific). Samples were shielded from light and incubated for 20 minutes at room temperature.

A BD LSRFortessa flow cytometer and FACSDiva software (RRID: SCR_001456) were used to process samples. FSC-A and SSC-A plots were produced, and single-cell populations were identified. A total of 10,000 events were recorded per sample. Negative unstained controls were run alongside experimental samples. FCS files were analyzed using open-source Floreada.io software (RRID: SCR_025286) and GraphPad Prism version 10.2.3 (RRID: SCR_002798). Cell-cycle histograms are representative of three independent experiments, and quantification combines three independent experiments. Fold change calculated versus control, error bars represent SEM, and statistical significance was determined using one-way ANOVA with *P* ≤ 0.05.

### Live cell imaging

U2OS H2B-GFP/mCherry-α-tubulin cells were incubated overnight in an Ibidi 8-well chamber slide. Immediately before live cell imaging, the medium was replaced with compounds dissolved in CM. Cells were imaged at 5-minute intervals for 24 hours on a Zeiss Axio Observer using a Plan Apochromat 20×/0.8 M27 air objective while being maintained at 37°C in an atmosphere of 5% CO_2_. For mitotic duration and mitotic outcome quantifications, 10 cells were analyzed per field of view with two fields per condition for each experiment. Images were analyzed using ImageJ software (RRID: SCR_003070). Duration and outcome were assessed independently on different randomly selected cells. Mitotic duration was calculated from cell round-up until either two daughter cells were produced or mitotic slippage occurred, if the movie ended prior to either outcome, this is indicated on the graph (Fig. 2A). Mitotic outcome was assessed morphologically. Plots combine two independent experiments.

### IF microscopy

MCF7 cells were plated on high-precision 1.5H 12 mm coverslips (Marienfeld) in 24-well plates in 1 mL CM. For tubulin experiments, coverslips were treated for 24 hours with compounds in CM.

For microtubule (MT)–kinetochore attachment experiments, coverslips were treated with palbociclib 1 μmol/L for 24 hours, washed once with PBS, then every 30 minutes for six washes with CM, and allowed to recover for 16 hours. CM was replaced with RO3306 10 μmol/L in CM for 2 hours before three quick CM washes. After 15 minutes, when mitotic cells were seen, MG132 10 μmol/L in CM was added for 30 minutes. Compounds in CM with MG132 10 μmol/L were added for 10 minutes.

Cells were fixed in 4% paraformaldehyde in PBS for 15 minutes at room temperature. Following three PBS washes, coverslips were blocked in 1 mL 3% BSA in PBS with 0.5% Triton-X100 for 30 minutes at room temperature. Coverslips were incubated in primary antibody overnight at 4°C. After three PBS washes, coverslips were incubated in a secondary antibody cocktail with DAPI for 2 hours at room temperature. Following three PBS washes, coverslips were dipped in absolute ethanol, air-dried, and mounted with ProLong antifade reagent (Molecular Probes). Coverslips were imaged on a DeltaVision 100×/1.40 NA U Plan S Apochromat objective and analyzed using ImageJ.

For tubulin IF experiments, 10 mitotic cells were imaged per coverslip; images are representative of three independent experiments. For MT–kinetochore experiments, CENPC was used to identify kinetochores, and MAD1 positivity was used to assess kinetochore attachment. Images are representative of three independent experiments. Quantification represents MAD1-positive kinetochores from five mitotic cells per condition and combines three independent experiments.

### Live cell and IF imaging statistical analysis

Figure 2 graphs (A, E) are plotted as violin plots using PlotsOfData (https://huygens.science.uva.nl/PlotsOfData/; ref. 20). This allows the spread of data to be accurately visualized along with the 95% confidence intervals (thick vertical bars) calculated around the median (thin horizontal lines) to allow statistical comparison between all treatments and timepoints. When the vertical bar of one condition does not overlap with one in another condition, the difference between the medians is statistically significant (*P* < 0.05); error bars represent SD.

### *In vitro* MT growth assay

Dynamic MTs were generated and observed as described previously (21). Briefly, purified tubulin proteins (biotinylated tubulin, rhodamine-labeled tubulin, florescence HiLyte 488 tubulin, and unlabeled porcine tubulin) were sourced from Cytoskeleton. To prepare MT seeds, a 20 μmol/L porcine tubulin mix containing 18% biotinylated tubulin, 12% rhodamine-labeled tubulin, and 70% unlabeled tubulin was incubated with 1 mmol/L GMPCPP (Jena Bioscience, NU-405S) first on ice and then at 37°C for 30 minutes. To separate the MTs from non-polymerized tubulins, centrifugation was performed using an Airfuge (Beckman Coulter) for 5 minutes. The MTs underwent another cycle of depolymerization and polymerization with 1 mmol/L GMPCPP. Finally, the MT seed samples were stored in MRB80 buffer (80 mmol/L PIPES (Sigma Aldrich P6757), pH 6.8, 1 mmol/L MgCl2, and 1 mmol/L EGTA) with 10% glycerol.

Coverslips were plasma cleaned using a Carbon Coater (Agar Scientific) and treated with PlusOne Repel-Silane (GE Healthcare) for 10 to 15 minutes. They were then cleaned further by sonication in methanol and rinsed with ultrapure water. Flow chambers were constructed using the cleaned coverslips and microscopy slides.

The chambers were treated with 0.2 mg/mL PLL-PEG-biotin (Surface Solutions) in MRB80 buffer for 5 minutes. After washing with MRB80 buffer, 1% pf127 was flowed through the chamber and incubated for 5 minutes, followed by washing the chamber with MRB80 buffer. Rhodamine-labeled MT seeds were incubated with 1 mg/mL NeutrAvidin (Thermo Fisher Scientific) for 5 minutes. MT seeds were attached to the coverslips with biotin–NeutrAvidin links. Finally, the chambers were incubated with 1 mg/mL κ-casein.

The *in vitro* reaction mixture was prepared in MRB80 buffer, consisting of 12 μmol/L tubulin mix (11.5 μmol/L unlabeled tubulin and 0.5 μmol/L fluorescent HiLyte 488 tubulin), 50 mmol/L KCl, 2 mmol/L MgCl_2_, 0.1% methylcellulose, 0.5 mg/mL κ-casein, 1 mmol/L GTP, 6 mmol/L DTT, and oxygen scavenging system (400 μg/mL glucose-oxidase, 200 μg/mL catalase, 4 mmol/L DTT, and 20 mmol/L glucose). The mixture was centrifuged for 5 minutes using Airfuge at room temperature. The supernatant was then mixed with nocodazole and elraglusib of indicated concentrations and added to the flow chamber containing MT seeds. The chamber was sealed with vacuum grease and observed at 30°C with TIRF microscopy.

Images of dynamic MTs were acquired by TIRF microscopy with a Nikon Eclipse Ti-E inverted research microscope equipped with diode lasers (405, 488, 561, and 647 nm; Coherent), Acousto-Optic Tunable Filters shutter (Solamere Technology), appropriate filters (Chroma), perfect focus system, the CFI Apochromat TIRF 100× 1.49 N.A. oil objective lens (Nikon), Evolve Delta electron-multiplying charge-coupled device 512 × 512 camera (Photometrics), and NIS-Elements AR software.

Images were analyzed using ImageJ. Minor imaging drift was corrected using the HyperStackRg plugin, and kymographs were generated in time sequence along a chosen line for an individual MT using the Multi Kymograph plugin on ImageJ. MT growth speeds were calculated by measuring the change in MT growth (0.16 μm per pixel) over time. Catastrophe frequency (events per minute) was determined by dividing the total number of catastrophe events by the total time MTs spent growing. Analysis was performed using GraphPad Prism version 10.2.3 with one-way ANOVA, with *P* ≤ 0.05 considered statistically significant.

### Cell lysates

#### Tubulin

Tubulin fractionation was achieved using a method adapted from Gundersen and colleagues (22). MT stabilization buffer (MSB) was made in dH_2_O [85 mmol/L PIPES (Sigma-Aldrich P6757), 1 mmol/L EGTA, 1 mmol/L MgCl_2_, 2 mol/L glycerol, 0.5% Triton-X100, and one cOmplete, mini, EDTA-free protease inhibitor cocktail tablet (Merck) per 10 mL].

Suspension cells were plated at four million cells/well in six-well plates and treated for 5 hours. Cells were pelleted, washed in PBS, and resuspended in 200 μL MSB at room temperature. Lysates were centrifuged at 12,000 rpm for 15 minutes. Supernatants were transferred to fresh tubes with 50 μL 5× Laemmli’s SDS loading buffer [10% w/v SDS, 250 mmol/L Tris-HCl, 50% v/v glycerol, 0.025% (w/v) bromophenol-blue, and 5% (v/v) β-mercaptoethanol] and represent the soluble fraction. A total of 250 μL of 1× Laemmli’s SDS loading buffer was added to the lysate pellet representing the insoluble fraction.

Adherent cells were plated at 500,000 cells/well in six-well plates and treated for 5 hours. Following a PBS wash, 200 μL of MSB was added to each well. Cells were scraped with a rigid cell lifter, and the lysate was centrifuged as above. As an additional step to increase the yield of insoluble tubulin, 250 μL of 1× Laemmli’s SDS loading buffer was added to the insoluble debris remaining in each well of the six-well plates and incubated at room temperature for 15 minutes. Following centrifugation, the soluble fractions were obtained from supernatant as above. The 1× Laemmli’s buffer from the wells was added to the lysate debris pellet to obtain the insoluble fraction.

Soluble and insoluble lysate samples were heated to 95°C for 15 minutes at 750 rpm on a thermoshaker and stored at −20°C. Each well was loaded with 10 µL of each sample onto acrylamide gels.

#### PARP/γH2AX

PARP cleavage was used to assess for apoptosis and γH2AX for DNA damage by immunoblot. Cell lysates were prepared for SDS-PAGE as previously described, and protein was quantified by Bradford assay (Bio-Rad; ref. 23).

#### Immunoblots

Tubulin, GSK3, β-Catenin, and PARP samples were run on 10% SDS-PAGE in tris-glycine running buffer (25 mmol/L Tris, 192 mmol/L glycine, and 0.1% SDS), and γH2AX samples were run on 15% SDS-PAGE gels in MES buffer (Thermo Fisher Scientific). Proteins were transferred onto nitrocellulose membranes, blocked for 1 hour in 1% BSA in TBS-T [40 mmol/L Tris and 150 mmol/L NaCl with 0.05% (v/v) Tween 20], and incubated overnight at 4°C in primary antibody. Membranes were washed with TBS-T, incubated in secondary antibody for 1 hour, washed in TBS-T, and imaged. Blot analysis was on the LI-COR Odyssey scanner with quantification on ImageStudio Lite 5.2. Blots are representative of three independent experiments; quantification combines three independent experiments with normalization to actin loading control and to experimental control. Analysis was performed using GraphPad Prism version 10.2.3 with one-way ANOVA, with *P* ≤ 0.05 considered statistically significant.

### Data availability

The datasets used and/or analyzed during the current study are available from the corresponding authors on request.

## Results

### Elraglusib induces a mitotic arrest in lymphoma cells

**Figure 1:**
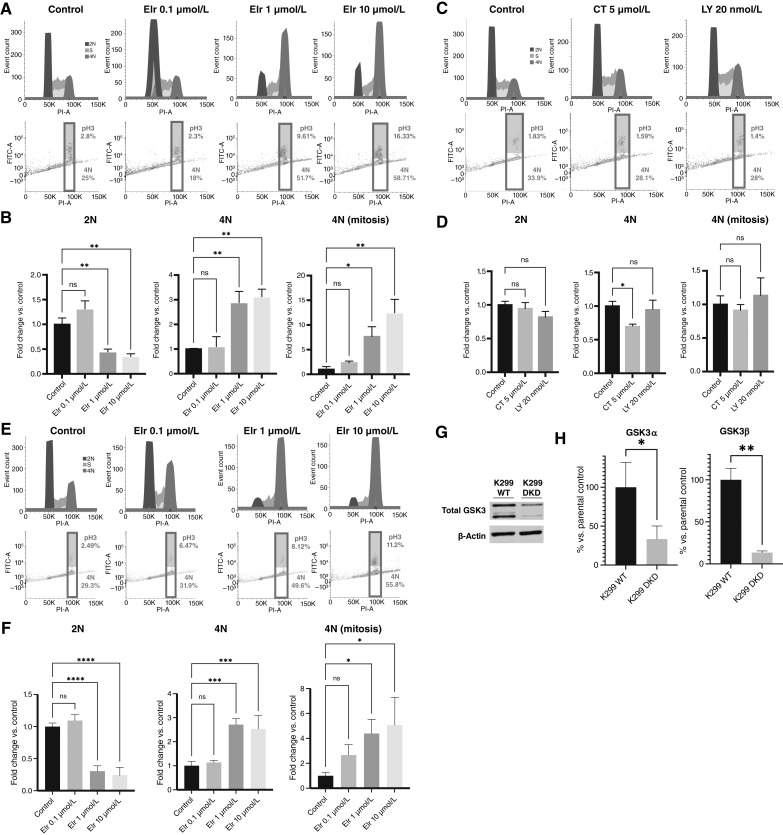
Elraglusib causes a mitotic arrest. **A** and **B,** Example flow cytometry plots (**A**) and quantifications (**B**) showing the effect of 24-hour elraglusib (Elr) treatment on the percentage of 2N, 4N, and 4N-mitotic K299 WT cells. **C** and **D,** Example flow cytometry plots (**C**) and quantifications (**D**) following 24-hour treatments with two structurally distinct GSK3 inhibitors, CT99021 and LY2090314 in K299 WT cells. **E** and **F,** Example flow cytometry plots (**E**) and quantifications (**F**) showing the effect of 24-hour elraglusib (Elr) treatment on the percentage of 2N, 4N, and 4N-mitotic K299 *GSK3A/B* dual paralog shRNA knockdown cells (DKD). **G** and **H,** Total GSK3 immunoblot (**G**) with quantification confirming knockdown efficiency in K299 DKD compared with K299 WT cells (**H**). Cells were treated for 24 hours prior to cell-cycle analysis by flow cytometry using propidium iodide and anti–phospho-histone-H3 (Ser10) staining. Ten thousand events were analyzed per sample. Cell-cycle histograms are representative of three independent experiments, and quantifications combine three independent experiments. Immunoblot representative of three independent experiments; quantification was performed by normalizing to actin loading control and combines three independent experiments. Fold change calculated vs. control, error bars represent SEM, and statistical significance was determined using one-way ANOVA, with *P* ≤ 0.05. ns, not significant; *, *P* < 0.05; **, *P* < 0.01; ***, *P* < 0.001; ****, *P* < 0.0001.

Elraglusib has been reported to induce cell-cycle arrest at G2 and/or M phases in cancer cell lines based upon cellular DNA content analysis using propidium iodide (6, 9, 10). Therefore, we sought to investigate these effects further. To distinguish between a G2 (4N) and M-phase (mitotic 4N) arrest, we performed flow cytometry using propidium iodide and anti–phospho-histone H3 Ser10 (H3-pS10) staining. Elraglusib treatment for 24 hours in K299 lymphoma cells led to a dose-dependent accumulation of H3-pS10–positive mitotic cells, and, to a lesser extent, 4N cells that are H3-pS10–negative (Fig. 1A and B). Statistically significant changes were observed at elraglusib concentrations at or above 1 μmol/L. These are clinically relevant doses because phase I trial data show elraglusib reaches peak plasma concentration around 17 to 20 μmol/L and remains above 1 μmol/L for 24 hours post-infusion (14). Similar experiments with two structurally distinct GSK3 inhibitors, CT99021 and LY2090314, produced no cell-cycle arrest in either cell line (Fig. 1C and D) at concentrations shown to inhibit GSK3 activity to a greater extent than that achieved by elraglusib (16). To confirm that the effect of elraglusib was occurring independently of GSK3, we also analyzed dual paralog *GSK3A/B* shRNA knockdown K299 cells (Fig. 1E and F) and, despite significant reduction in both GSK3α and GSK3β (Fig. 1G and H), confirmed a similar accumulation of mitotic H3-pS10 cells to that seen in the WT cells (Fig. 1A and B). We conclude that elraglusib primarily causes a mitotic arrest and that these effects are not due to inhibition of GSK3.

### Elraglusib impairs kinetochore–MT attachment to cause a mitotic arrest

**Figure 2:**
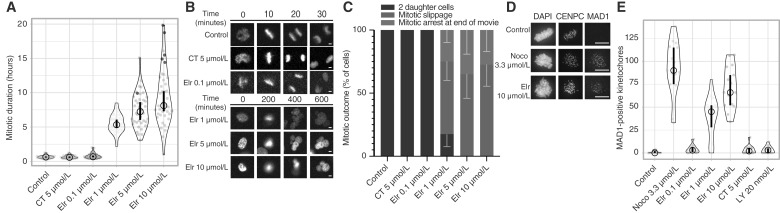
Elraglusib impairs kinetochore–MT attachment to cause mitotic arrest and mitotic slippage (**A–C**). Mitotic duration (**A**) with CT99021 (CT) and elraglusib (Elr), example images (**B**), and mitotic fate quantifications (**C**). U2OS H2B-GFP/mCherry-α-tubulin cells were treated and imaged every 5 minutes for 24 hours. Ten cells were analyzed per field of view, with two fields per condition for each experiment. Mitotic duration (**A**) and fate plots (**C**) combine two independent experiments. For graph **A**, red data points indicate the movie ended with the cell remaining in mitosis, and the thick vertical lines represent a 95% confidence interval around the median, which can be used for statistical comparison of treatments by eye (see “Materials and Methods”). For graph **C**, error bars represent SD. **D** and **E,** Example images (**D**), number of MAD1-positive kinetochores with nocodazole (Noco), elraglusib (Elr), CT99021 (CT), and LY2090314 (LY); (**E**). MCF7 cells were synchronized in G1 with palbociclib for 24 hours and then released for 16 hours in CM before a 2-hour RO3306 block, wash off and MG132 treatment for 30 minutes. Cells were treated for 10 minutes with nocodazole (Noco), elraglusib (Elr), CT99021 (CT), or LY209014 (LY) before fixing and IF microscopy. CENPC was used to identify kinetochores, and MAD1 positivity was used to assess kinetochore attachment. Five mitotic cells were imaged per coverslip. Images are representative of three independent experiments. Quantification represents MAD1-positive kinetochores from five mitotic cells per condition and combines three independent experiments. For graph **E**, the thick vertical lines represent a 95% confidence interval around the median, which can be used for statistical comparison of treatments by eye (see “Materials and Methods”). Scale bars, 10 μm.

To understand why elraglusib causes a mitotic arrest, we performed live cell imaging of U2OS cells expressing H2B-GFP. Although control cells or GSK3-inhibited cells completed mitosis within a typical 30-minute timeframe, cells treated with elraglusib (at ≥1 μmol/L) were delayed in mitosis for many hours (Fig. 2A). This mitotic delay was associated with a failure to align chromosomes correctly [Fig. 2B; Supplementary Movies S1 (elraglusib 1 μmol/L) and Supplementary Movies S2 (DMSO 0.1% control)]. Cells remained arrested in this state until the end of the movie, or they exited mitosis with unaligned chromosomes, in a process known as mitotic slippage (Fig. 2B and C). Mitotic slippage is frequently observed following MT-based therapies, leading to apoptosis or senescence in G1 (24).

We hypothesized that these defects were due to an inability to properly attach chromosomes to MTs via the kinetochore. In this situation, the spindle assembly checkpoint (SAC) is activated to delay mitotic exit (25). This is due to the presence of MAD1 on unattached kinetochores, which generates the SAC signal until kinetochores become attached to MTs and MAD1 is removed. IF imaging demonstrated that 10 minutes of elraglusib treatment significantly increased the number of unattached, MAD1-positive kinetochores in MCF7 cells (Fig. 2D and E). Fully depolymerizing MTs with 3.3 μmol/L nocodazole (26) also increased MAD1 kinetochore occupancy to near 100%, as expected; however, GSK3 inhibition with CT99021 or LY2090314 had no effect. We conclude that elraglusib delays chromosome alignment by inhibiting kinetochore–MT attachment, leading to a persistent mitotic arrest that eventually results in mitotic slippage. These effects cannot be explained by inhibition of GSK3.

### Elraglusib acts by directly destabilizing MTs

**Figure 3:**
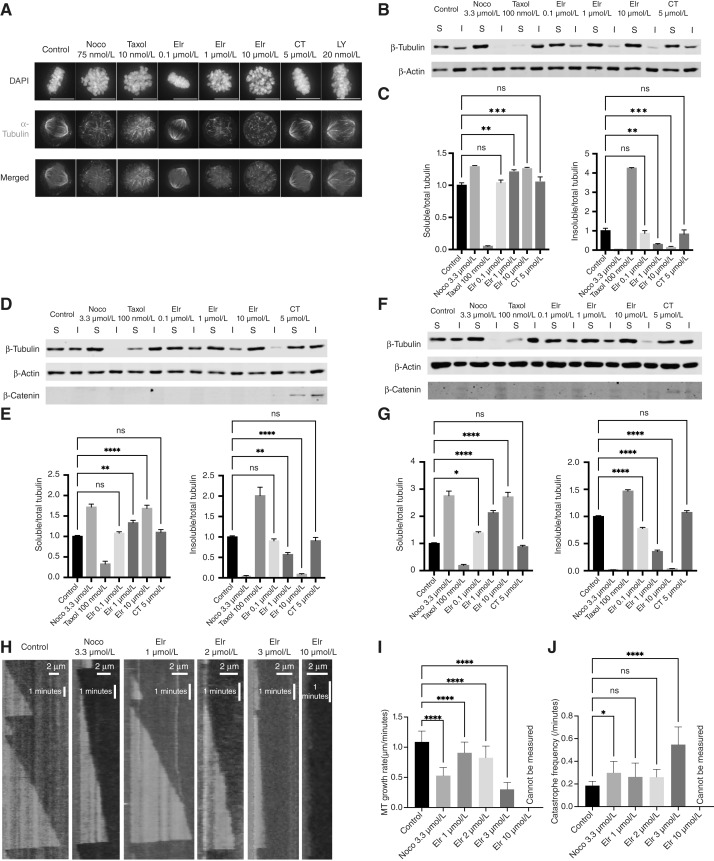
Elraglusib acts via direct MT destabilization (**A–J**). Example images (**A**) of MCF7 cells treated with nocodazole (Noco), paclitaxel (Taxol), elraglusib (Elr), and CT99021 (CT). MCF7 cells were treated for 24 hours before fixation and IF microscopy. Ten mitotic cells were imaged per coverslip; images are representative of three independent experiments. Tubulin fractionation experiments comparing soluble (S) and insoluble (I) tubulin by immunoblot in MCF7 (**B** and **C**), K299 (**D** and **E**) and HH (**F** and **G**) cells. Cells were treated for 5 hours and lysed in MT stabilization buffer before fractionation into soluble (S) and insoluble (I) tubulin and analysis by immunoblot. Quantification was performed by normalizing to actin loading control and then to the experimental control. Blots are representative of three independent experiments, and quantification combines three independent experiments. Fold change calculated vs. control, error bars represent SEM, and statistical significance was determined using one-way ANOVA, with *P* ≤ 0.05. Scale bars, 10 μm. **H,** Kymographs illustrating dynamics of MT growth *in vitro* in the absence of drug (control) or the presence of 3.3 μmol/L nocodazole, 1, 2, 3, or 10 μmol/L elraglusib. Rhodamine-labeled short stable MTs were seeded on the coverslip, and 12 μmol/L tubulins (containing 4% fluorescent HiLyte 488 tubulins) were added to the reaction to generate and visualize dynamic MTs. Scale bars, 2 μm. **I** and **J,** Quantification of MT growth rate (**I**) and catastrophe frequency (**J**) with no drug treatment (control) or with 3.3 μmol/L nocodazole, 1, 2, 3 or 10 μmol/L elraglusib. The number of analyzed MT growth events: *n* = 34 for control; *n* = 42 for 3.3 μmol/L nocodazole; and *n* = 43, 24, and 21 for 1, 2, and 3 μmol/L elraglusib, respectively. MT catastrophe frequency was analyzed for dynamic MT on individual MT seeds–the number of analyzed MT seeds: *n* = 13 for control; *n* = 20 for 3.3 μmol/L nocodazole; and *n* = 13, 18, and 15 for 1, 2, and 3 μmol/L elraglusib, respectively. Error bars represent SD. Statistical significance was determined using one-way ANOVA, with *P* ≤ 0.05. ns, not significant; *, *P* < 0.05; **, *P* < 0.01; ***, *P* < 0.001; ****, *P* < 0.0001.

Kinetochore–MT attachment errors could be due to changes at the kinetochore and/or the MTs. To assess the latter, we performed IF imaging of mitotic MCF7 cells treated for 24 hours with elraglusib (Fig. 3A). This demonstrated defects in mitotic spindle assembly, similar to the effects observed with low doses of nocodazole, implying MT polymerization could be inhibited. To probe this further, we performed tubulin fractionation experiments to directly quantify the proportion of polymerized and depolymerized MTs, which appear in the insoluble and soluble fractions, respectively (22). Figure 3B and C demonstrate that treatment of MCF7 cells with elraglusib for 5 hours caused a dose-dependent decrease in insoluble tubulin and a corresponding increase in soluble tubulin. This was similar to the effects observed with the MT depolymerizer nocodazole, but opposite to those observed with the MT stabilizer paclitaxel (Fig. 3B and C). Similar effects were also observed in K299 (Fig. 3D and E) and HH cells (Fig. 3F and G).

In almost all cases, elraglusib affects MT solubility at or above 1 μmol/L, which is consistent with the concentrations needed to cause mitotic delays and unattached kinetochores. We also observed no effect on MT solubility with the GSK3 inhibitor CT99021 at 5 μmol/L, despite the fact that the downstream GSK3 target β-catenin was stabilized at this concentration, indicating significant GSK3 inhibition (Fig. 3B–G; ref. 2).

To test whether elraglusib directly induces MT destabilization independently of MT-associated proteins or motor proteins, we performed *in vitro* MT growth assays (Fig. 3H–J). Elraglusib impaired MT growth in a dose-dependent manner (Fig. 3I) and increased the frequency of MT catastrophe (Fig. 3J). These effects were similar to those observed with nocodazole. No clear MTs were observed at the highest concentration of elraglusib used (10 μmol/L), suggesting significant tubulin depolymerization.

### Elraglusib-induced genotoxicity and apoptosis are abolished by a cell-cycle arrest

**Figure 4:**
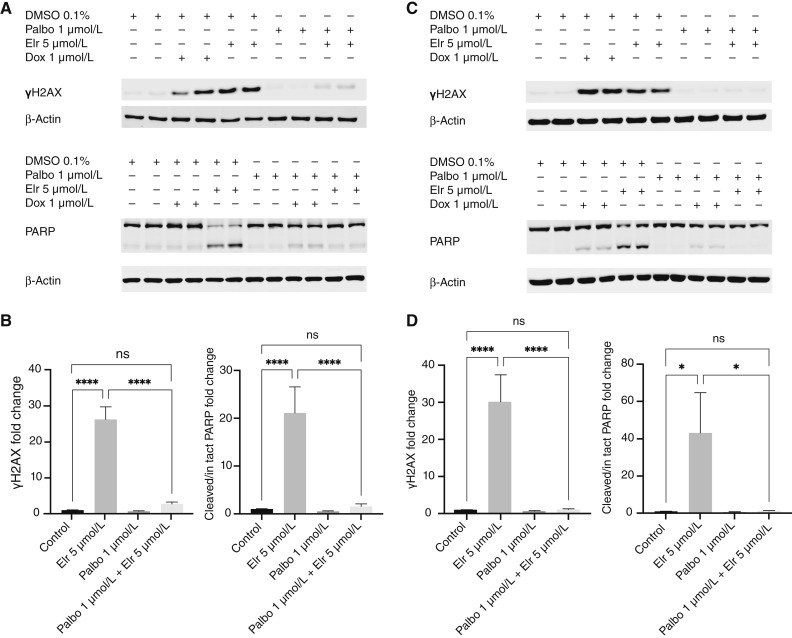
Elraglusib-induced genotoxicity and cytotoxicity are abolished by cell-cycle arrest (**A–D**). K299 (**A** and **B**), and HH cells (**C** and **D**) were arrested in palbociclib (Palbo; or treated with DMSO control) for 24 hours before the addition of elraglusib (Elr), doxorubicin (Dox), or DMSO for a further 24 hours. Cells were then lysed and analyzed by immunoblot. PARP cleavage was used for apoptosis assessment and γH2AX for DNA damage. Quantification was performed by normalizing to actin and the experimental control. Blots are representative of three independent experiments, and quantification combines three independent experiments. Fold change calculated vs. control, error bars represent SEM, and statistical significance was determined using one-way ANOVA, with *P* ≤ 0.05. ns, not significant; *, *P* < 0.05; ****, *P* < 0.0001.

Mitotic slippage commonly leads to DNA damage and apoptosis (24). We hypothesized that this could explain the cytotoxicity observed following elraglusib treatment. In agreement, elraglusib treatment led to a significant increase in γH2AX and induced PARP cleavage in both K299 (Fig. 4A and B) and HH cells (Fig. 4C and D), compared with DMSO-treated controls. These levels were similar to or higher than observed following treatment with the DNA-damaging agent doxorubicin. Importantly, pretreatment with palbociclib 1 μmol/L for 24 hours to arrest cells in G1 prior to elraglusib treatment essentially abolished these effects (27). This demonstrates that the genotoxic effects of elraglusib require cell-cycle progression, most likely because cells must transit through mitosis and undergo mitotic slippage to promote apoptosis.

### Elraglusib impairs the proliferation of primary human T cells

**Figure 5:**
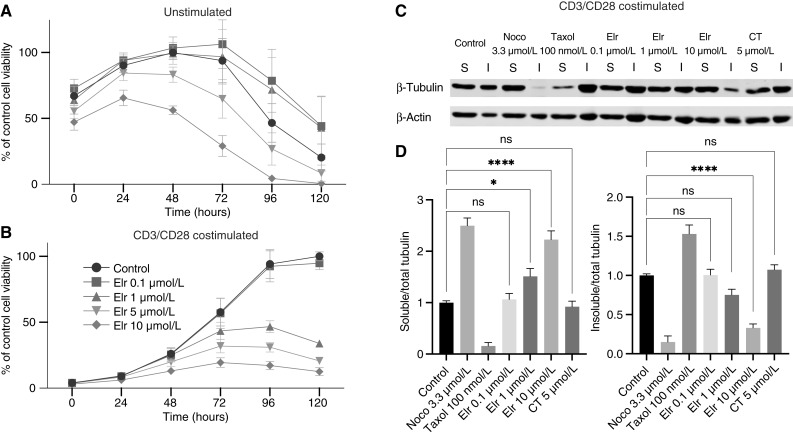
Elraglusib impairs proliferation and reduces cell viability in stimulated primary human T cells but does not affect unstimulated cells (**A** and **B**). Primary human T cells were isolated from PBMCs from healthy volunteers. Unstimulated controls (**A**) remained in CM, and stimulated cells (**B**) were treated with a CD3/CD28-co-stimulation kit. Cells were treated with DMSO control or elraglusib (Elr). Cell viability was assayed using Promega RealTime-Glo MT assay and the plate read daily for 120 hours. Data combine three independent experiments with T cells from three individual volunteers. Data normalized to the experimental control peak luminescence. Elraglusib increases the soluble tubulin fraction and decreases the insoluble tubulin fraction in stimulated primary human T cells (**C** and **D**). Proliferating stimulated primary human T cells were treated for 5 hours and assayed for soluble and insoluble tubulin as described above. Blots are representative of three independent experiments, and quantification combines three independent experiments. Fold change calculated vs. control, error bars represent SEM, and statistical significance was determined using one-way ANOVA, with *P* ≤ 0.05. ns, not significant; *, *P* < 0.05; ****, *P* < 0.0001.

Elraglusib produces cytotoxic effects in T-cell lymphoma lines (6, 16). Therefore, we sought to compare if these effects were also observed in primary T cells. Figure 5 shows that the viability of unstimulated primary human T cells was not affected by elraglusib at concentrations of 0.1, 1, or 5 μmol/L but was reduced by 10 μmol/L (Fig. 5A). In contrast, primary human T cells activated and stimulated by CD3/CD28 co-stimulation were affected by elraglusib in a dose-dependent manner at concentrations at or above 1 μmol/L (Fig. 5B). Tubulin fractionation immunoblots performed on proliferating primary human T cells showed that elraglusib led to an increase in soluble tubulin fraction and a reduction in the insoluble tubulin fraction at similar concentrations that induced cytotoxicity (≥1 μmol/L; Fig. 5C and D). CT99021 5 μmol/L had no significant effect on soluble or insoluble tubulin fractions in these cells. We conclude that elraglusib affects primary human T cells at similar concentrations to lymphoma lines. These effects require T-cell activation, consistent with proliferation being needed to drive cells through mitosis and induce mitotic slippage.

## Discussion

Elraglusib was developed as a targeted GSK3β inhibitor, and its anticancer effects have been variously attributed to GSK3 inhibition within cancer cells, resulting in abnormal regulation of NF-κB, impaired DNA damage responses, or myc downregulation, and to immunomodulatory effects within NK and T cells (7, 13, 28, 29). We, and others, have reported that the drug inhibits both the α and β paralogs of GSK3, along with several other kinases in cell-free assays (10, 16). We also showed that other validated small molecule GSK3α/β inhibitors do not share the anti-lymphoma properties of elraglusib and that *GSK3A/B* shRNA knockdown did not affect the elraglusib IC_50_ (16, 17). Preprint data from Cosgun and colleagues (bioRxiv 2023.03.13.532152) also report broad cytotoxic effects of elraglusib in the absence of β-catenin or myc stabilization, suggesting it occurs without significant GSK3 inhibition (16). Together, these findings question the requirement for GSK3 inhibition in the mechanism of action of elraglusib.

We report here that elraglusib causes a mitotic arrest in various different cancer lines, consistent with the G2 or M arrest observed previously in lymphoma, renal, and bladder cancer lines (6, 9, 10). Importantly, this arrest is associated with direct MT depolymerization which prevents chromosomal alignment leading to mitotic slippage, DNA damage, and apoptosis. The process of mitotic slippage is likely to explain the significant increase in nonmitotic H3-pS10–negative 4N cells that appear in the cell-cycle analysis of elraglusib-treated cells (Fig. 1). It is also likely to be crucial for the resulting DNA damage and apoptosis because the genotoxic effects of elraglusib are abolished by prior inhibition of CDK4/6 to prevent cell-cycle progression.

Similar effects are commonly observed with other anticancer agents that block MT polymerization, such as the alkaloids vincristine and vinblastine, and nocodazole (30–32). A recent genome-wide CRISPR/Cas9 knockout screen identified that aurora kinase A knockout or inhibition sensitizes pancreatic cancer cell lines to elraglusib (33). Interestingly, aurora kinase A inhibition has previously been shown to synergize with vincristine, a recognized MT destabilizer, suggesting a shared mechanism of action (34).

The ability of elraglusib to induce mitotic slippage, DNA damage, and apoptosis suggests that it acts similarly to a range of other antimitotic drugs that delay mitosis by activating the SAC. These include MT stabilizers, such as paclitaxel (31, 35), inhibitors of MT motors, such as Eg5 (36, 37), or inhibitors of mitotic kinases, such as PLK1 (38). These drugs all cause side effects due to off-target effects on proliferating healthy cells, especially within the hematopoietic precursor cell compartment, which limits their clinical tolerability. So, could elraglusib offer benefits over commonly used antimitotic drugs?

Recently presented trial data from patients with pancreatic cancer treated with elraglusib show a correlation between treatment-related neutropenia and survival, suggesting that off-target effects on healthy cells are probably inevitable to achieve good treatment responses (39). Elraglusib has been reported to specifically target lymphoma cells without affecting the viability of unstimulated normal B or T lymphocytes, which could in theory help lymphocytes to mount an antitumor immune response (6). However, our data suggest that this difference is due to a lower rate of proliferation in unstimulated lymphocytes, because stimulating the proliferation of primary T lymphocytes enhances elraglusib toxicity (Fig. 5) and preventing proliferation of lymphoma lines abolished cytotoxicity (Fig. 4). The *in vitro* CD3/CD28 co-stimulation model we utilized to stimulate T lymphocytes is designed to mimic T-cell responses to antigen-presenting cells *in vivo*, suggesting that elraglusib may have immunosuppressive effects in patients (40). However, it is important to note that elraglusib was recently shown to enhance immune cell activation and synergize with anti–PD-L1 therapy in a mouse model of colorectal cancer (28). Whether these effects are due to direct effects on the immune system or enhanced immune engagement due to cytotoxic effects on colorectal cancer cells remains to be determined.

An interesting clinical observation that seems to separate elraglusib from other MT-based drugs concerns the relative lack of neuronal side effects. MT poisons affect axonal transport leading to chemotherapy-induced peripheral neuropathy, and these are frequent dose-limiting toxicities for both vinca alkaloids and taxanes (41). Therefore, it is noteworthy that neuropathy has not yet emerged as a significant side effect of elraglusib (14). Whether this is due to a unique mechanism of action, early-phase data with small patient cohorts, poorer tubulin engagement, or a dosing effect is unclear currently and requires further evaluation.

Further work will be required to establish the elraglusib tubulin-binding site. Tumor specimens should also be used to confirm that a mitotic arrest phenotype is occurring *in vivo* and identify intratumoral drug concentrations in animal models and humans.

Elraglusib is not unique in its ability to inhibit both kinases and tubulin polymerization. Tivantinib was developed as a MET inhibitor, received FDA orphan status, and underwent a phase III clinical trial for the treatment of hepatocellular carcinoma (42). Its mechanism does not rely on MET inhibition but depends upon its ability to act as a MT destabilizing agent (43, 44). Rigosertib, initially identified as a PLK1 inhibitor, but then proposed to function as a RAS mimetic, has also been found to act as a MT destabilizer (45–48). Crystallography confirmed that the compound binds tubulin, and specific β-tubulin mutations induce rigosertib resistance without conferring resistance to vinblastine. Conversely, nocodazole, which is not used clinically, is widely used as a MT destabilizing compound but also inhibits several kinases, including ABL, c-KIT, BRAF, and MEK (49).

Off-target effects frequently contribute to the mechanism of action of cancer drugs in clinical trials, and incorrectly attributing the mechanism may confound patient selection, leading to misinterpretation of results and increasing the risk of trial failure (50). Altering a candidate compound to optimize pharmacodynamic and pharmacokinetic properties is often required as part of lead optimization, and maintaining target engagement requires knowledge of the relevant target (51). Similarly, the identification of a predictive biomarker, which relies upon detailed biological understanding, significantly increases the chance of clinical trial success and FDA approval (52). A detailed understanding of drug mechanisms is also required to appreciate the therapeutic selective pressures which drive resistance, a common cause of treatment failure (53).

Early-phase trial evidence demonstrates the potential clinical efficacy of elraglusib in pancreatic cancer, melanoma, and adult T-cell leukemia/lymphoma (14, 15). Our work suggests that these anticancer effects occur via MT destabilization rather than GSK3 inhibition. This has implications for the development of GSK3 expression or activity assays as predictive biomarkers for elraglusib response, the evaluation of potential drug resistance mechanisms, the assessment of anticipated patient toxicities, and the design of synergistic drug combinations in future clinical trials.

### Conclusion

Elraglusib acts as a direct MT destabilizer both *in vitro* and across cell types, resulting in mitotic arrest, genotoxicity, and apoptosis. These effects account for its broad pan-cancer cytotoxicity, which does not rely upon GSK3 inhibition and cannot be replicated by other GSK3 inhibitors. These mechanistic data are important for future compound development, clinical trial design, patient toxicity assessment, and predictive biomarker development, which should not focus on GSK3-related mechanisms alone.

## Supplementary Material

Supplementary Movie 1 Supplementary Movie 1

Supplementary Movie 2 Supplementary Movie 2

## References

[1] Embi N , Rylatt DB, Cohen P. Glycogen synthase kinase-3 from rabbit skeletal muscle. Separation from cyclic-AMP-dependent protein kinase and phosphorylase kinase. Eur J Biochem 1980;107:519–27. 6249596

[2] Sutherland C . What are the bona fide GSK3 Substrates? Int J Alzheimers Dis 2011;2011:505607. 21629754 10.4061/2011/505607 PMC3100594

[3] Jope RS , Johnson GVW. The glamour and gloom of glycogen synthase kinase-3. Trends Biochem Sci 2004;29:95–102. 15102436 10.1016/j.tibs.2003.12.004

[4] Frame S , Cohen P, Biondi RM. A common phosphate binding site explains the unique substrate specificity of GSK3 and its inactivation by phosphorylation. Mol Cell 2001;7:1321–7. 11430833 10.1016/s1097-2765(01)00253-2

[5] Quintayo MA , Munro AF, Thomas J, Kunkler IH, Jack W, Kerr GR, . GSK3β and cyclin D1 expression predicts outcome in early breast cancer patients. Breast Cancer Res Treat 2012;136:161–8. 22976805 10.1007/s10549-012-2229-8

[6] Wu X , Stenson M, Abeykoon J, Nowakowski K, Zhang L, Lawson J, . Targeting glycogen synthase kinase 3 for therapeutic benefit in lymphoma. Blood 2019;134:363–73. 31101621 10.1182/blood.2018874560 PMC6659256

[7] Ding L , Madamsetty VS, Kiers S, Alekhina O, Ugolkov A, Dube J, . Glycogen synthase kinase-3 inhibition sensitizes pancreatic cancer cells to chemotherapy by abrogating the TopBP1/ATR-mediated DNA damage response. Clin Cancer Res 2019;25:6452–62. 31533931 10.1158/1078-0432.CCR-19-0799 PMC6825568

[8] Hilliard TS , Gaisina IN, Muehlbauer AG, Gaisin AM, Gallier F, Burdette JE. Glycogen synthase kinase 3 beta inhibitors induce apoptosis in ovarian cancer cells and inhibit in-vivo tumor growth. Anticancer Drugs 2011;22:978–85. 21878813 10.1097/CAD.0b013e32834ac8fc PMC3188381

[9] Kuroki H , Anraku T, Kazama A, Bilim V, Tasaki M, Schmitt D, . 9-ING-41, a small molecule inhibitor of GSK-3beta, potentiates the effects of anticancer therapeutics in bladder cancer. Sci Rep 2019;9:19977. 31882719 10.1038/s41598-019-56461-4 PMC6934761

[10] Pal K , Cao Y, Gaisina IN, Bhattacharya S, Dutta SK, Wang EF, . Inhibition of GSK-3 induces differentiation and impaired glucose metabolism in renal cancer. Mol Cancer Ther 2014;13:285–96. 24327518 10.1158/1535-7163.MCT-13-0681 PMC3956125

[11] Ugolkov A , Gaisina I, Zhang J-S, Billadeau DD, White K, Kozikowski A, . GSK-3 inhibition overcomes chemoresistance in human breast cancer. Cancer Lett 2016;380:384–92. 27424289 10.1016/j.canlet.2016.07.006 PMC5786372

[12] Ugolkov A , Qiang W, Bondarenko G, Procissi D, Gaisina I, James CD, . Combination treatment with the GSK-3 inhibitor 9-ING-41 and CCNU cures orthotopic chemoresistant glioblastoma in patient-derived xenograft models. Transl Oncol 2017;10:669–78. 28672195 10.1016/j.tranon.2017.06.003 PMC5496477

[13] Karmali R , Chukkapalli V, Gordon LI, Borgia JA, Ugolkov A, Mazar AP, . GSK-3β inhibitor, 9-ING-41, reduces cell viability and halts proliferation of B-cell lymphoma cell lines as a single agent and in combination with novel agents. Oncotarget 2017;8:114924–34. 29383130 10.18632/oncotarget.22414 PMC5777742

[14] Carneiro BA , Cavalcante L, Mahalingam D, Saeed A, Safran H, Ma WW, . Phase I study of elraglusib (9-ING-41), a glycogen synthase kinase-3β inhibitor, as monotherapy or combined with chemotherapy in patients with advanced malignancies. Clin Cancer Res 2024;30:522–31. 37982822 10.1158/1078-0432.CCR-23-1916

[15] Hsu A , Huntington KE, De Souza A, Zhou L, Olszewski AJ, Makwana NP, . Clinical activity of 9-ING-41, a small molecule selective glycogen synthase kinase-3 beta (GSK-3β) inhibitor, in refractory adult T-Cell leukemia/lymphoma. Cancer Biol Ther 2022;23:417–23. 35815408 10.1080/15384047.2022.2088984 PMC9272832

[16] Coats JT , Tauro S, Sutherland C. Elraglusib (formerly 9-ING-41) possesses potent anti-lymphoma properties which cannot be attributed to GSK3 inhibition. Cell Commun Signal 2023;21:131. 37316860 10.1186/s12964-023-01147-8 PMC10265916

[17] Coats JT , Tauro S, Sutherland C. Redundancy of glycogen synthase kinase 3 in lymphoma cell viability, proliferation, and the cytotoxicity of elraglusib. Blood 2023;142(Suppl 1):5802.

[18] Finlay D , Patel S, Dickson LM, Shpiro N, Marquez R, Rhodes CJ, . Glycogen synthase kinase-3 regulates IGFBP-1 gene transcription through the thymine-rich insulin response element. BMC Mol Biol 2004;5:15. 15350195 10.1186/1471-2199-5-15 PMC517930

[19] Voets E , Marsman J, Demmers J, Beijersbergen R, Wolthuis R. The lethal response to Cdk1 inhibition depends on sister chromatid alignment errors generated by KIF4 and isoform 1 of PRC1. Sci Rep 2015;5:14798. 26423135 10.1038/srep14798 PMC4589785

[20] Postma M , Goedhart J. PlotsOfData-A web app for visualizing data together with their summaries. PLoS Biol 2019;17:e3000202. 30917112 10.1371/journal.pbio.3000202 PMC6453475

[21] Doodhi H , Kasciukovic T, Clayton L, Tanaka TU. Aurora B switches relative strength of kinetochore-microtubule attachment modes for error correction. J Cell Biol 2021;220:e202011117. 33851957 10.1083/jcb.202011117 PMC8050843

[22] Gundersen GG , Khawaja S, Bulinski JC. Postpolymerization detyrosination of alpha-tubulin: a mechanism for subcellular differentiation of microtubules. J Cell Biol 1987;105:251–64. 2886509 10.1083/jcb.105.1.251 PMC2114889

[23] Rena G , Guo S, Cichy SC, Unterman TG, Cohen P. Phosphorylation of the transcription factor forkhead family member FKHR by protein kinase B. J Biol Chem 1999;274:17179–83. 10358075 10.1074/jbc.274.24.17179

[24] Cheng B , Crasta K. Consequences of mitotic slippage for antimicrotubule drug therapy. Endocr Relat Cancer 2017;24:T97–106. 28684541 10.1530/ERC-17-0147

[25] Lara-Gonzalez P , Pines J, Desai A. Spindle assembly checkpoint activation and silencing at kinetochores. Semin Cell Dev Biol 2021;117:86–98. 34210579 10.1016/j.semcdb.2021.06.009 PMC8406419

[26] Brito DA , Yang Z, Rieder CL. Microtubules do not promote mitotic slippage when the spindle assembly checkpoint cannot be satisfied. J Cell Biol 2008;182:623–9. 18710927 10.1083/jcb.200805072 PMC2518701

[27] Foy R , Lew KX, Saurin AT. The search for CDK4/6 inhibitor biomarkers has been hampered by inappropriate proliferation assays. NPJ Breast Cancer 2024;10:19. 38438376 10.1038/s41523-024-00624-8 PMC10912267

[28] Huntington KE , Louie AD, Srinivasan PR, Schorl C, Lu S, Silverberg D, . GSK-3 inhibitor elraglusib enhances tumor-infiltrating immune cell activation in tumor biopsies and synergizes with anti-PD-L1 in a murine model of colorectal cancer. Int J Mol Sci 2023;24:10870. 37446056 10.3390/ijms241310870 PMC10342141

[29] Shaw G , Cavalcante L, Giles FJ, Taylor A. Elraglusib (9-ING-41), a selective small-molecule inhibitor of glycogen synthase kinase-3 beta, reduces expression of immune checkpoint molecules PD-1, TIGIT and LAG-3 and enhances CD8^+^ T cell cytolytic killing of melanoma cells. J Hematol Oncol 2022;15:134. 36104795 10.1186/s13045-022-01352-x PMC9472445

[30] Jordan MA , Thrower D, Wilson L. Mechanism of inhibition of cell proliferation by Vinca alkaloids. Cancer Res 1991;51:2212–22. 2009540

[31] Schiff PB , Horwitz SB. Taxol stabilizes microtubules in mouse fibroblast cells. Proc Natl Acad Sci U S A 1980;77:1561–5. 6103535 10.1073/pnas.77.3.1561 PMC348536

[32] Xu K , Schwarz PM, Ludueña R. Interaction of nocodazole with tubulin isotypes. Drug Development Res 2002;55:91–6.

[33] Ding L , Maeder E, Zhang C, Weiskittel T, Schmitt D, Mazar A, . Abstract 4658: genome wide CRISPR/Cas9 library screening identifies aurora kinase A as a regulator of elraglusib sensitivity in pancreatic cancer. Cancer Res 2024;84(Suppl 6):4658.

[34] Lentini L , Amato A, Schillaci T, Insalaco L, Di Leonardo A. Aurora-A transcriptional silencing and vincristine treatment show a synergistic effect in human tumor cells. Oncol Res 2008;17:115–25. 18669163 10.3727/096504008785055521

[35] Sudo T , Nitta M, Saya H, Ueno NT. Dependence of paclitaxel sensitivity on a functional spindle assembly checkpoint. Cancer Res 2004;64:2502–8. 15059905 10.1158/0008-5472.can-03-2013

[36] Mayer TU , Kapoor TM, Haggarty SJ, King RW, Schreiber SL, Mitchison TJ. Small molecule inhibitor of mitotic spindle bipolarity identified in a phenotype-based screen. Science 1999;286:971–4. 10542155 10.1126/science.286.5441.971

[37] DeBonis S , Skoufias DA, Lebeau L, Lopez R, Robin G, Margolis RL, . In vitro screening for inhibitors of the human mitotic kinesin Eg5 with antimitotic and antitumor activities. Mol Cancer Ther 2004;3:1079–90. 15367702

[38] Lénárt P , Petronczki M, Steegmaier M, Di Fiore B, Lipp JJ, Hoffmann M, . The small-molecule inhibitor BI 2536 reveals novel insights into mitotic roles of polo-like kinase 1. Curr Biol 2007;17:304–15. 17291761 10.1016/j.cub.2006.12.046

[39] Ugolkov A , Koukol A, Kellinger C, Smith SL, Gallipoli S, Gagnon S, . Correlation of therapy-induced neutropenia with survival in patients with metastatic pancreatic cancer treated with GSK-3 inhibitor elraglusib (9-ING-41) in combination with gemcitabine/nab-paclitaxel in the 1801 phase 2 study. J Clin Oncol 2024;42(Suppl 16):e16311.

[40] Trickett A , Kwan YL. T cell stimulation and expansion using anti-CD3/CD28 beads. J Immunol Methods 2003;275:251–5. 12667688 10.1016/s0022-1759(03)00010-3

[41] Nicolini G , Monfrini M, Scuteri A. Axonal transport impairment in chemotherapy-induced peripheral neuropathy. Toxics 2015;3:322–41. 29051467 10.3390/toxics3030322 PMC5606679

[42] Rimassa L , Assenat E, Peck-Radosavljevic M, Pracht M, Zagonel V, Mathurin P, . Tivantinib for second-line treatment of MET-high, advanced hepatocellular carcinoma (METIV-HCC): a final analysis of a phase 3, randomised, placebo-controlled study. Lancet Oncol 2018;19:682–93. 29625879 10.1016/S1470-2045(18)30146-3

[43] Xiang Q , Zhen Z, Deng DY, Wang J, Chen Y, Li J, . Tivantinib induces G2/M arrest and apoptosis by disrupting tubulin polymerization in hepatocellular carcinoma. J Exp Clin Cancer Res 2015;34:118. 26458953 10.1186/s13046-015-0238-2 PMC4603939

[44] Aoyama A , Katayama R, Oh-Hara T, Sato S, Okuno Y, Fujita N. Tivantinib (ARQ 197) exhibits antitumor activity by directly interacting with tubulin and overcomes ABC transporter-mediated drug resistance. Mol Cancer Ther 2014;13:2978–90. 25313010 10.1158/1535-7163.MCT-14-0462

[45] Jost M , Chen Y, Gilbert LA, Horlbeck MA, Krenning L, Menchon G, . Combined CRISPRi/a-based chemical genetic screens reveal that rigosertib is a microtubule-destabilizing agent. Mol Cell 2017;68:210–23.e6. 28985505 10.1016/j.molcel.2017.09.012 PMC5640507

[46] Jost M , Chen Y, Gilbert LA, Horlbeck MA, Krenning L, Menchon G, . Pharmaceutical-grade rigosertib is a microtubule-destabilizing agent. Mol Cell 2020;79:191–8.e3. 32619469 10.1016/j.molcel.2020.06.008 PMC7332992

[47] Gumireddy K , Reddy MV, Cosenza SC, Boominathan R, Baker SJ, Papathi N, . ON01910, a non-ATP-competitive small molecule inhibitor of Plk1, is a potent anticancer agent. Cancer Cell 2005;7:275–86. 15766665 10.1016/j.ccr.2005.02.009

[48] Athuluri-Divakar SK , Vasquez-Del Carpio R, Dutta K, Baker SJ, Cosenza SC, Basu I, . A small molecule RAS-mimetic disrupts RAS association with effector proteins to block signaling. Cell 2016;165:643–55. 27104980 10.1016/j.cell.2016.03.045 PMC5006944

[49] Park H , Hong S, Hong S. Nocodazole is a high-affinity ligand for the cancer-related kinases ABL, c-KIT, BRAF, and MEK. ChemMedChem 2012;7:53–6. 22002881 10.1002/cmdc.201100410

[50] Lin A , Giuliano CJ, Palladino A, John KM, Abramowicz C, Yuan ML, . Off-target toxicity is a common mechanism of action of cancer drugs undergoing clinical trials. Sci Transl Med 2019;11:eaaw8412. 31511426 10.1126/scitranslmed.aaw8412 PMC7717492

[51] Hughes JP , Rees S, Kalindjian SB, Philpott KL. Principles of early drug discovery. Br J Pharmacol 2011;162:1239–49. 21091654 10.1111/j.1476-5381.2010.01127.x PMC3058157

[52] Wong CH , Siah KW, Lo AW. Estimation of clinical trial success rates and related parameters. Biostatistics 2019;20:273–86. 29394327 10.1093/biostatistics/kxx069 PMC6409418

[53] Vasan N , Baselga J, Hyman DM. A view on drug resistance in cancer. Nature 2019;575:299–309. 31723286 10.1038/s41586-019-1730-1 PMC8008476

